# Paraquat and Parkinson’s Disease: The Molecular Crosstalk of Upstream Signal Transduction Pathways Leading to Apoptosis

**DOI:** 10.2174/1570159X21666230126161524

**Published:** 2023-04-07

**Authors:** Wesley Zhi Chung See, Rakesh Naidu, Kim San Tang

**Affiliations:** 1 Jeffrey Cheah School of Medicine and Health Sciences, Monash University Malaysia, 47500 Bandar Sunway, Selangor, Malaysia;; 2 School of Pharmacy, Monash University Malaysia, 47500 Bandar Sunway, Selangor, Malaysia

**Keywords:** Apoptosis, catenins, mitogen-activated protein kinases, paraquat, Parkinson’s disease, phosphatidylinositol 3-kinase, proto-oncogene proteins c-akt, TOR serine-threonine kinases

## Abstract

Parkinson’s disease (PD) is a heterogeneous disease involving a complex interaction between genes and the environment that affects various cellular pathways and neural networks. Several studies have suggested that environmental factors such as exposure to herbicides, pesticides, heavy metals, and other organic pollutants are significant risk factors for the development of PD. Among the herbicides, paraquat has been commonly used, although it has been banned in many countries due to its acute toxicity. Although the direct causational relationship between paraquat exposure and PD has not been established, paraquat has been demonstrated to cause the degeneration of dopaminergic neurons in the substantia nigra *pars compacta*. The underlying mechanisms of the dopaminergic lesion are primarily driven by the generation of reactive oxygen species, decrease in antioxidant enzyme levels, neuroinflammation, mitochondrial dysfunction, and ER stress, leading to a cascade of molecular crosstalks that result in the initiation of apoptosis. This review critically analyses the crucial upstream molecular pathways of the apoptotic cascade involved in paraquat neurotoxicity, including mitogen-activated protein kinase (MAPK), phosphatidylinositol-4,5-bisphosphate 3-kinase (PI3K)/AKT, mammalian target of rapamycin (mTOR), and Wnt/β-catenin signaling pathways.

## 1 INTRODUCTION

Parkinson’s disease (PD) is the second most common and progressive neurodegenerative movement disorder after Alzheimer’s disease, affecting 1% of the population older than the age of 65 worldwide [1]. PD is characterized by tremors, bradykinesia, rigidity, and postural instability, in addition to other non-motor characteristics manifesting in the form of cognitive and neuropsychiatric symptoms, such as cognitive deficits, dementia, anxiety, depression, and sleep disorders [2]. The motor features of PD are predominantly evident after approximately 70% of the dopaminergic neurons have been lost and are a consequence of the selective degeneration of the dopaminergic neurons located in the substantia nigra pars compacta [3]. However, it also may be due to the progressive loss of axons and synapses that result in reduced dopamine neurotransmission [3]. Pathologically, the presence of intraneuronal proteinaceous cytoplasmic inclusions known as Lewy bodies and Lewy neurites, along with the loss of dopaminergic neurons, is recognized as the main hallmark of PD [4]. The protein α-synuclein has been the primary component in Lewy pathology and is considered indispensable in the formation of Lewy bodies and Lewy neurites [5]. To date, there is still no cure for PD. Historically, dopaminergic therapies remained the gold standard for improving the motor function of PD patients by increasing extracellular dopamine concentration at the striatal regions. However, long-term dopamine-increasing treatments can lead to adverse effects such as dyskinesias, fluctuations, and loss of efficacy. Emerging therapies for PD have focused mainly on developing small-molecule drugs, gene therapies, and immunotherapies to target genes and proteins implicated in PD pathogenesis [6]. In addition, cellular therapies are currently being developed to slow the disease’s progression or replace dopaminergic neurons [6, 7].

To date, the exact cause of PD is yet to be deciphered. Nonetheless, PD is a multifactorial disorder with intertwined interactions between genes and the environment affecting various cellular pathways and neural networks resulting in the initiation and development of the disease [8]. Only approximately 10% of PD cases are attributed to genetic factors, while the remaining idiopathic cases are linked to other environmental contributors, such as exposure to pesticides, herbicides, and heavy metals [9, 10]. PD, regardless of whether it is the idiopathic or genetic origin, is phenotypically heterogeneous in terms of clinical progression and symptoms [11].

N,N'-Dimethyl-4,4'-bipyridinium dichloride, more commonly known as paraquat, is an important member of the bipyridylium family of broad-spectrum herbicides and has been linked to the development of PD [12]. In a study involving a sizeable agricultural cohort, AGRICAN (AGRIculture and CANcer), which was initiated in France to assess the relationship between agricultural exposures and cancer incidence and mortality, reported that an increased risk of PD was associated with farmers exposed to paraquat for one to 46 years [13]. Similarly, paraquat exposure in the age group of > 70 years contributes to approximately 21-24% of PD cases in Taiwan [14]. Since the introduction of paraquat to the market in the 1960s, fatality cases due to paraquat poisoning have increased [15]. Post-mortem investigation of the brains of eight patients who succumbed to paraquat poisoning showed cerebral changes, which include generalized edema, perivascular necrosis, hemorrhages, and neuroinflammation indicated by the activation of astrocyte and microglia [16]. Nonetheless, no studies showed a direct causational relationship between paraquat exposure and PD [17].

Paraquat has a similar molecular structure and biochemistry to 1-methyl-4-phenyl-1,2,3,6-tetrahydropyridine (MPTP), another common neurotoxin that has been demonstrated to reproduce parkinsonian features in cellular and animal PD models. Although both paraquat and MPTP exert their toxic effects *via* oxidative stress, paraquat elicits its harmful effects on dopaminergic neurons in a distinctive manner compared to MPTP [18, 19]. Paraquat penetrates the blood-brain barrier into the brain through a neutral amino acid transporter and subsequently is transported into the neuronal cell in a sodium-dependent fashion [20]. Paraquat belongs to a class of redox cycling compounds that involves a process of alternate reduction and reoxidation, resulting in the generation of reactive oxygen species (ROS) and reactive nitrogen species (RNS), including hydrogen peroxide (H_2_O_2_), superoxide ion (O_2_^•-^), and peroxynitrite ion (ONOO^-^) [21-23].

Dopaminergic neurons are vulnerable to paraquat toxicity as these neurons express dopamine transporter (DAT). The monovalent cation form of paraquat, PQ^+^, is transported into dopaminergic neurons by DAT, which induces oxidative damage [24]. Paraquat has been observed to increase lipid peroxidation, impair mitochondrial function, increase α- synuclein expression and aggregation, and decrease the level of antioxidants such as glutathione and neuroinflammation, subsequently promoting apoptosis of dopaminergic neurons [25-28]. In this review, we will provide an overview of apoptosis and recapitulate evidence of paraquat to activate the apoptotic signaling pathway. Subsequently, we will focus on the upstream pathways of apoptosis affected by paraquat. The pharmacological effects of paraquat are summarised in Table **1**.

## 2 ACTIVATION OF APOPTOSIS SIGNALING PATHWAY

Apoptosis is the key mechanism of neuronal cell death in PD. The relevance of apoptosis in neurodegeneration, especially in PD, and its associated events have been extensively elaborated in our previous work [29]. The cell death process is characterized by changes in cellular morphology, such as cell shrinkage, chromatin condensation, nuclear fragmentation, and plasma membrane blebbing [30]. Apoptosis is vital in innumerable physiological processes in development and aging to maintain or eliminate undesired and superfluous cell populations in tissues [31]. During the development and maturation of the neurons, apoptosis is essential in shaping the nervous system and developing appropriate neuronal circuitry [32]. The initiation of apoptosis is a tightly regulated process mediated by an intracellular proteolytic cascade of proteases known as caspases [33]. Caspases are widely synthesized in the cells as procaspases, which are activated by proteolytic cleavage at the aspartic acid residues by another member of the caspase family, thereby amplifying the proteolytic cascade [33]. Apoptotic caspases consist of initiator caspases (caspase-8 and -9) that function to initiate the apoptotic machinery and executioner caspases (caspase-3, -6, and -7) that degrade cellular components by mass proteolysis [34].

Upstream of caspases, the BCL-2 family of intracellular proteins primarily regulates apoptosis by direct physical protein-protein interactions that modulate mitochondrial outer membrane permeabilization (MOMP). The BCL-2 family is divided into three groups; (1) anti-apoptotic proteins [*i.e*., BCL-2, BCL-X_L_, myeloid cell leukemia-1 (MCL-1)], (2) pro-apoptotic pore formers (*i.e*., BAX and BAK), and (3) pro-apoptotic BH3-only proteins (*i.e*., BAD, Noxa, BNIP3) [35]. Regulation of the balance between these pro- and anti-apoptotic factors of the BCL-2 family is pivotal for determining whether the cell undergoes survival or death [33]. When the cells are committed to apoptosis, BH3-only proteins bind to the mitochondrial outer membrane, increasing the binding affinity of BAX and BAK [36]. This will result in the oligomerization and insertion of BAX and BAK into the mitochondrial bilayer to form pores or MOMP [35]. Subsequently, the pro-apoptotic intermembrane space proteins such as cytochrome c, apoptosis-inducing factor (AIF), and Smac/DIABLO are released from the mitochondria into the cytosol resulting in the recruitment and activation of caspases [37].

Apoptosis can be activated by extrinsic and intrinsic pathological stimuli, including ROS and RNS in the case of paraquat exposure [33]. Innumerable *in vitro* and murine models demonstrated neurotoxicity related to the exposure of paraquat conciliated by ROS-dependent apoptosis [23, 38-41]. In addition, paraquat neurotoxicity has been observed to be mediated *via* a BAK-dependent mechanism involving BH3-only members, Noxa and BNIP3, which act upstream of BAK [42]. BAK is constitutively expressed on the mitochondrial surface, where it is inhibited by pro-survival proteins such as MCL-1, BCL-2, and BCL-X_L_ [43]. The binding of Noxa to MCL-1 and BNIP3 to BCL-2/BCL-X_L_ causes the disinhibition of BAK and activation of MOPS [44-46]. In a study performed by Fei *et al.* [42], exposure of SK-N-SH cells to paraquat showed upregulation in the mRNA and protein expression of BAK, BNIP3, and Noxa, in addition to cytochrome c and cleaved caspase-3 levels, indicating the activation of the apoptotic cascade. Apart from cytochrome c, paraquat has also been demonstrated to release other pro-apoptotic intermembrane space proteins, such as HtrA2/Omi and Smac/DIABLO, but not AIF and endonuclease G [28, 38].

## 3 MITOGEN-ACTIVATED PROTEIN KINASE (MAPK) PATHWAY

The MAPK pathway is one of the oldest and evolutionally conserved families of serine/threonine protein kinases which plays a role in regulating cellular processes such as proliferation, stress response, immune defense, and apoptosis [47]. The signals from various extracellular stimuli are facilitated through a cascade of events in the cell, which phosphorylates and alters a myriad of substrate activities in the nucleus, cytoplasm, mitochondria, Golgi apparatus, and endoplasmic reticulum. MAPK signaling cascade includes an operating set of three succedent evolutionarily conserved groups of protein kinase comprised of MAPK, mitogen-activated protein kinase kinase (MAP2K), and mitogen-activated protein kinase kinase kinase (MAP3K) [48]. The activation of MAPK cascades occurs in a module of sequential phosphorylation of downstream MAPK, *i.e*., phosphorylation of MAP3K, followed by the phosphorylation of MAP2K, which in turn, activates MAPK, such as c-Jun N-terminal kinases (JNK), p38, or extracellular signal-regulated kinase (ERK) [49].

In mammalian cells, three well-known MAPK pathways have been described: JNK, p38, and ERK1/2 [50]. All three MAPK pathways are implicated in *in vitro* and *in vivo* models of PD using paraquat. Niso-Santano *et al.* [51] analyzed the protein expression of apoptosis signal-regulating kinase 1 (ASK1), a member of the MAP3K family, which is activated in response to various stimuli and relays signals to the stress-activated protein kinase (SAPK). The study concluded a concentration-dependent decrease of p-ASK1Ser83 and p-ASK1Ser967, in addition to an increase in p-ASK1Thr845 protein expression upon exposure of SH-SY5Y cells to paraquat (25 to 100 μM, 24 hours). This confirms that paraquat promoted the ASK1 activation by dephosphorylating Ser83 and Ser967, and phosphorylating Thr845 residues. Activation of ASK1 has been linked to ER stress and the initiation of apoptosis and autophagy in several neurodegenerative diseases, including PD [52]. This was demonstrated by Niso-Santano *et al.* [53], where the protein expression of phosphorylated inositol-requiring transmembrane kinase/ endoribonuclease 1 (IRE1), a key activator of the unfolded protein response (UPR), was substantially higher in paraquat-treated SH-SY5Y cells overexpressing wild-type ASK1 when compared to untransfected cells treated with the herbicide.

JNK has been widely known as one of the SAPKs based on their activation in response to a wide range of different stress factors, including inflammatory cytokines [54], oxidative stress [55], and DNA damage [56]. The JNK pathway contributes to the control of cellular processes, including those involved in regulating cell proliferation, differentiation, and apoptosis [57]. The role of JNK in cell proliferation and apoptosis is dependent on the cell type and stimuli involved [58, 59]. JNK can be activated when upstream protein kinases of MAP2K family, including mitogen-activated protein kinase kinase (MKK)4 and MKK7, phosphorylates the Tyr183 and Thr185 residues of JNK [60]. MKK7 is essential for JNK activation upon stimulation by stresses, whereas MKK4 is required for optimal JNK activation [60]. The phosphorylation of JNK causes the translocation of JNK in the cytosol into the nucleus resulting in the activation of different transcriptional factors such as c-Jun and p53 tumor suppressor protein [61]. A concentration-dependent increase in protein expression of p-MKK4/7, p-JNK, and p-c-Jun in SH-SY5Y cells was observed upon exposure to 25-100 μM paraquat for 24 hours, indicating the activation of the JNK pathway [51, 62].

In cells undergoing stress, JNK-mediated phosphorylation can stabilize and activate p53 to initiate apoptotic cell death [63]. This was confirmed in a study by Niso-Santano *et al.* [51], where a significant increase in the percentage of cells undergoing apoptosis, indicated by increased levels of diffuse cytochrome c and active caspase-3 in JNK-activated SH-SY5Y cells treated with paraquat. In addition, pre-treatment of the JNK inhibitor SP600125 before paraquat treatment attenuates cell death [51]. It has been reported in an *in vivo* Drosophila model that mRNA and protein expression levels of JNK and caspase-3 were increased in the brain upon exposure to paraquat [23, 41].

Another SAPK, p38, was detected in neurons with or without Lewy bodies located in the substantia niga pars compacta (SNpc) of post-mortem brain samples [64]. p38 is activated by the upstream protein kinases MAP2K family, *i.e*., MKK3 and MKK6 [65, 66]. These two MAP2K kinases are highly selective for p38 and do not activate JNK or ERK [67]. Niso-Santano *et al.* [51] demonstrated a concentration-dependent increase in the p-MKK3/6 protein expression in SH-SY5Y cells upon exposure to paraquat (25-100 μM, 24 hours). Similar to JNK, p38 also phosphorylates p53, which promotes the translocation to the nucleus, resulting in gene transcription of pro-apoptotic mediators such as BAX to initiate the apoptotic machinery [68]. This can be seen in a study by Ju *et al.* [62], where increased protein expression of p-38 (~1.4-fold), p53 (~1.5-fold), and apoptotic mediators such as BAX/BCL-2 ratio, caspase-3, and caspase-9 were observed in SH-SY5Y cells treated with paraquat (300 μM, 24 hours).

Another signaling cassette that is central to the MAPK pathway is ERK. In contrast to JNK and p38, which are activated primarily by the stress response, activation of the ERK cascade has always been thought to inhibit apoptosis in response to mitogenic stimuli leading to the production of proteins required for cell growth and differentiation [69]. Exposure of SH-SY5Y cells to paraquat (300 μM, 24 hours) has been observed to downregulate the protein expression of p-ERK by 1.3-fold, leading to an increased proportion of cells undergoing apoptosis [62]. Nonetheless, an increase in protein expression of ERK1/2 and cytochrome C release has been demonstrated in mouse NIH3T3 embryonic fibroblasts treated with paraquat, indicating such differential effects of the ERK pathway could reflect cell-type specificity [70]. Another study conducted by Niso-Santano *et al.* [71] using rat E18 neuroblasts concluded low concentration of paraquat increases protein expression of p-ERK1/2 from the very early beginning of 2.5 to 10 minutes, with the maximal protein expression of p-ERK1/2 at 5 minutes. This indicates that time could play an essential role in the protein expression of ERK. A moderate ERK immunoreactivity was observed in the Lewy body of the SNpc and other nuclei of the brain stem from post-mortem PD brain samples [64]. Moreover, SNpc neurons from post-mortem brain samples displayed unusual coarse, discrete, granular accumulation of p-ERK in the cytoplasm and mitochondria, which were absent in the age-matched control group [72, 73].

## 4 PHOSPHATIDYLINOSITOL-4,5-BISPHOSPHATE 3-KINASE (PI3K)/AKT SIGNALING PATHWAY

Upon its discovery in the 1980s, PI3K/AKT intracellular signal transduction pathway has been involved in regulating multiple cellular processes such as cell proliferation, differentiation, survival, growth, and metabolism [74]. The PI3K/ AKT pathway consists of two components: PI3K and its serine/threonine downstream molecule, AKT [75]. As a major downstream molecule of receptor tyrosine kinase (RTK) and G protein-coupled receptors, PI3K functions to transduce signals from various neurotrophic factors, hormones, and cytokines into intracellular messages by generating phospholipids, which in turn activates AKT and various other extracellular matrix molecules and cytokines [74]. One of the well-known neurotrophic and anti-apoptotic pro-survival factors is insulin‐like growth factor 1 (IGF-1) [76]. The binding of IGF-1 to its corresponding RTK receptor, IGF-R1, triggers the downstream PI3K/AKT pathway that promotes cell proliferation [77]. Upon activation, AKT then translocates from the plasma membrane to the cytoplasm or nucleus, phosphorylating its target proteins. Accumulating evidence has strongly suggested that activation of the PI3K/AKT pathway promotes the survival and growth of dopaminergic neurons by inhibiting apoptosis and hence being dysregulated in PD patients [50, 78]. Post-mortem investigation of dopaminergic neurons in PD patients showed diminished levels of p-AKT [79]. In addition, a recent study showed that AKT and p-AKT are significantly reduced in the SNpc of PD patients [80]. Ju *et al.* [62] demonstrated a decrease in the protein expression of the p-IGF-1 receptors by 25% upon exposure of SH-SY5Y cells to paraquat (300 μM, 24 hours), resulting in a decrease in cell viability by 25%. Moreover, a reduction in p-PI3K and p-AKT protein expression by 40% and 10%, respectively, were also observed in the study.

As the primary molecule downstream of the PI3K signaling pathway, AKT has been shown to inhibit apoptosis by negatively regulating the function and expression of pro-apoptotic proteins and processes [81]. Several mechanisms have been elucidated by which AKT intervenes in the apoptotic cascade to promote cell survival. One of the most well-studied mechanisms is the direct phosphorylation of pro-apoptotic proteins such as BAD, BAX, and caspase-9 by AKT, resulting in the inhibition of the apoptosis cascade [82-84]. BAD protein is a pro-apoptotic member of the BCL-2 family, which plays a role in initiating apoptosis. When non-phosphorylated, the BAD protein mediates apoptosis by selectively dimerizing anti-apoptotic members of the same family, such as BCL-X_L_ and BCL-2, resulting in the displacement of the pro-apoptotic protein BAX [85, 86]. This allows BAX to be free to initiate mitochondrial membrane permeability and recruitment of caspases to initiate apoptosis. When phosphorylated by AKT, BAD cannot dimerize with anti-apoptotic BCL-X_L_ or BCL-2; hence apoptosis is inhibited [87]. Furthermore, AKT directly inhibits the conformational change of BAX and its subsequent translocation to mitochondria resulting in the inhibition of cytochrome c release [88]. Another mechanism by which AKT inhibits apoptosis is by directly maintaining the mitochondrial integrity to inhibit the release of cytochrome c from mitochondria [89]. Ju *et al.* [62] reported downregulation of AKT protein expression contributes to the initiation of apoptosis in SH-SY5Y cells treated with paraquat. Indeed, apoptotic markers such as BAD, BAX/BCL-2, caspase-9, and cytochrome c protein expression levels were increased significantly [62]. The downregulation of AKT might also be due to the crosstalk between AKT and p53 pathways. Under conditions leading to an irreversible apoptotic commitment, such as oxidative stress, p53 activation contributes to the initiation of apoptosis by inhibiting AKT [90]. An increase in the protein expression of p53 was also observed [62].

Another target protein of AKT is Forkhead box transcription factors of the class O (FOXO), an evolutionally conserved transcription factor family that plays a significant role in many cellular processes, such as proliferation, differentiation, and survival, in addition to mediating DNA repair and apoptosis [91]. FOXO controls cell proliferation and survival by regulating the expression of genes involved in the cell-cycle progression and apoptotic pathway. FOXO is present in the cytosol and nucleus, and its activity has been reported to function differently according to its upstream regulator, post-translational modification, and cellular environment [92]. Phosphorylation of FOXO by AKT can inhibit apoptosis by sequestration from the nucleus away from the death-inducing genes to the cytoplasm to be degraded by the ubiquitin-proteasome pathway [93]. However, the downregulation of AKT in *in vitro* and *in vivo* studies pertaining to the use of paraquat suggests that FOXO can be phosphorylated by other proteins. Accumulating evidence suggests that phosphorylation of FOXO by JNK enhances the translocation of FOXO to the nucleus and increases its activity to activate pro-apoptotic genes [94]. Indeed, paraquat increased the expression of p-JNK, p-FOXO, and cleaved caspase-3 in the brain of Drosophila flies [23].

Growing functional evidence has suggested a close functional relationship between AKT and MAPK cascades, in which increased phosphorylation activity of AKT has been shown to suppress the JNK/p38 SAPK pathway in many cell systems, thus inhibiting apoptosis [81]. This can be confirmed in a study by Ju *et al.* [62], where a decrease in the phosphorylated AKT was accompanied by an increase in p-JNK and p-p38 upon exposure of SH-SY5Y cells to paraquat (300 μM, 24 hours). Moreover, it has been demonstrated that AKT regulates ASK1 since ASK1 contains an AKT-specific phosphorylation site [95]. Therefore, ASK1 is one of the convergence points between PI3K/AKT and SAPK/MAPK cascades.

## 5 MAMMALIAN TARGET OF RAPAMYCIN (MTOR) SIGNALING PATHWAY

mTOR is a highly conserved serine/threonine protein kinase that belongs to the member of the PI3K-associated kinase protein family. mTOR is expressed in most mammalian cell types overseeing multiple functions, including cell survival, metabolism, and cytoskeletal organization [96]. mTOR functions in two multiprotein complexes with distinct subunit composition and regulation of downstream targets. mTORC1 lies downstream of AKT and is activated by p-AKT [74]. mTORC1 responds to signaling from glucose, amino acid, and growth factors, which promotes protein and lipid synthesis, in addition to nucleotide biogenesis required for cell proliferation and maintenance [97]. On the contrary, mTORC2 lies upstream of AKT and is regulated strictly under the control of growth factors such as insulin [98]. mTORC2 functions to phosphorylate and activate AKT to promote cell survival [97]. Both mTOR1 and mTOR2 signaling pathway is activated by extracellular and intracellular cues when conditions are favorable for proliferation and growth.

However, a growing body of evidence has highlighted the role of ER stress-induced activation of mTORC1 resulting in the reduced phosphorylation of AKT as a negative-feedback mechanism and, subsequently, induction of the IRE1-JNK pathway and apoptosis [99, 100]. Moreover, induction of ER stress also results in the phosphorylation of the mTORC2 subunit, rapamycin-insensitive companion of mammalian target of rapamycin (RICTOR), by glycogen synthase kinase-3 (GSK-3)β, which suppresses AKT activation [101]. The downregulation of AKT induced by mTORC2 phosphorylation also provides a positive feedback loop to mTORC1 for the induction of the IRE1-JNK pathway and, subsequently, apoptotic cell death [101].

In recent years, evidence has suggested the dysregulation of mTOR signaling in PD. Dijkstra *et al.* [102] studied the transcriptomic changes of the post-mortem SNpc of PD patients using microarray analysis and unsurprisingly observed the mRNA levels of mTORC1 and mTORC2 were upregulated even in the early pathological stages of PD and remained impaired in the later stages of the disease. Moreover, both mTOR and p-mTOR levels were upregulated in neurons with α-synuclein accumulation isolated from the post-mortem temporal cortex [103]. In line with these studies, paraquat has been demonstrated to increase the protein expression levels of p-GSK-3β and GSK-3β by 40% and 60%, respectively, in mouse striata [27]. In addition, the authors have concluded a 1.6-fold upregulation in the mTOR protein expression in the striata.

mTOR also plays a vital role in regulating mitochondrial dynamics and autophagy [104, 105]. Autophagy can promote cell survival by preventing cells from undergoing apoptosis. Impaired autophagy has been suggested to contribute to neuronal cell loss in PD [106]. mTOR signaling has been associated with the inhibition of autophagy [104]. It has been shown in a study using SH-SY5Y cells that paraquat-induced apoptosis was accelerated when autophagy was suppressed using autophagy inhibitor 3-methyladenine [107]. Mitochondrial damage plays an essential role in paraquat-induced cell death [108]. The inability to clear the dysfunctional mitochondria due to autophagy impairment has been linked to the development of PD [109].

## 6 WNT/β-CATENIN SIGNALING PATHWAY

The Wnt/β-catenin signaling pathway has emerged as one of the most important ancient and evolutionally conserved signaling pathways in the development and maintenance of physiological function in the adult brain [110]. The Wnt signaling pathway also significantly regulates different aspects of embryonic development, including cell fate determination, migration, polarity, neural patterning, and embryonic development [111]. In the absence of extracellular Wnt ligand binding to the seven-pass transmembrane Frizzled receptor and its associate co-receptor low-density lipoprotein-related receptors 5 and 6 (LRP5/6) at the plasma membrane, cytoplasmic β-catenin is phosphorylated by the components in the multiprotein destruction complex, such as casein kinase 1, GSK-3α, and GSK-3β [112]. This targets β-catenin for ubiquitination and subsequent proteolytic degradation by the proteasomal machinery. Upon binding of the Wnt ligand to its receptor complex inhibits the phosphorylation of β-catenin for destruction [110]. Subsequently, β-catenin accumulates in the cytoplasm and translocates to the nucleus. It activates specific transcription genes such as cyclin D1 and c-myc that are involved in cell proliferation, survival, differentiation, neurogenesis, and inflammation [113].

Recent findings have highlighted the prominent role of the Wnt/β-catenin signaling pathway in the development and maintenance of dopaminergic neurons [114-116]. A microarray study showed that β-catenin gene expression was downregulated in the SNpc of post-mortem PD patients [117]. β-catenin was also hypermethylated, in addition to reduced expression of the β-catenin gene [118]. Notably, the immunoreactivity of β-catenin was almost abolished in the nuclei of the PD SNpc dopaminergic neurons. Nevertheless, Yang *et al.* [119] demonstrated that the canonical Wnt/β-catenin signaling pathway was activated in paraquat-treated SH-SY5Y cells (300 μM, 24 hours), as evidenced by the time-dependent increase in β-catenin and p-GSK-3α and -3β. The phosphorylation of GSK-3 by proteins such as AKT causes inhibition of its kinase activity, thus, hampering the phosphorylation of β-catenin for proteasomal degradation [120-122]. However, as mentioned earlier, paraquat was shown to decrease AKT protein expression; therefore, phosphorylation of GSK-3 is unlikely to be mediated by the PI3K/AKT pathway. GSK-3 can phosphorylate LRP6, indicating the possibility of activating Frizzled and LRP co-receptors by paraquat [123]. In addition, ROS such as H_2_O_2_ has been demonstrated to activate the Wnt/β-catenin signaling pathway [124]. Furthermore, Bernkopf and Behrens [125] have reported the activation of the Wnt/β-catenin signaling pathway intrinsically *via* a signaling axis from mitochondria to β-catenin. Loss of ΔΨm in damaged or stressed mitochondria trigger the cleavage of mitochondrial phosphatase phosphoglycerate mutase 5 (PGAM5) by presenilin-associated rhomboid-like protein (PARL) [126]. The release of PGAM5 from the damaged mitochondria to the cytosol interacts with the multiprotein destruction complex, enhancing direct dephosphorylation of β-catenin and counteracting GSK3-mediated β-catenin phosphorylation [127]. Thus, the generation of ROS and the loss of ΔΨm upon exposure to paraquat may indirectly activate the Wnt/β-catenin signaling pathway.

The Wnt/β-catenin signaling pathway has also been linked to cell apoptosis [128]. Yang *et al.* [119] demonstrated the time-dependent increase in β-catenin and pro-apoptotic protein BAK, in addition to a decrease in cyclin D1 protein expression upon exposure of SH-SY5Y cells to PQ (300 μM, 24 hours). Cyclin D1 is a member of the cyclin protein family that positively regulates the G1/S transition – the only checkpoint in the cell cycle when cells can commit to another round of division or exit the cell cycle [129]. The expression of cyclin D1 is upregulated when growth factors and mitogens are present in the cellular environment [130]. β-catenin activates the transcription factor from the cyclin D1 promoter resulting in the expression of cyclin D1 to initiate the cell cycle progression [131]. However, Yang *et al.* [119] showed that the activation of β-catenin resulted in the inhibition of cyclin D1 expression, suggesting a crosstalk between the Wnt/β-catenin signaling and another important signaling pathway to regulate the expression of cyclin D. An increasing number of literature has demonstrated the involvement of the MAPK pathway in the regulation of cyclin D1 promoter [132]. Cyclin D1 promoter activity can be activated by the ERK cascade; however, it can also be inhibited by p38 and JNK pathways *via* various mechanisms [132]. Since paraquat has been reported to downregulate ERK and upregulate p38 and JNK protein expression, these mechanisms may be responsible for the downregulation of cyclin D. Thus, paraquat could cause dysregulation in the cell cycle by arresting the cells in the G1/S phase on top of initiating the apoptotic mechanism [133].

## CONCLUSION

Paraquat-induced neurotoxicity has been demonstrated to involve many cellular mechanisms, including oxidative stress, abnormal protein degradation and aggregation, altered dopamine catabolism, mitochondrial dysfunction, and ER stress that ultimately culminate in the initiation of apoptosis. However, the molecular targets in the proximal events leading to the core apoptotic machinery have yet to be described elsewhere. The synthesized review demonstrated the effect of paraquat in initiating apoptosis *via* different upstream crosstalk pathways, *i.e*., MAPK, PI3K/AKT, mTOR, and Wnt/β-catenin signaling pathways. The convergence points between the signaling transduction mechanisms are illustrated in Fig. (**1**). In conclusion, paraquat induces apoptotic cell death by activating MAPK, mTOR, and Wnt/β-catenin pathways while suppressing PI3K/AKT. The ability of paraquat to modulate the signaling molecules in mTOR and PI3K/AKT pathways reinforces the notion that autophagy and apoptosis are indeed interrelated. Deciphering the proximal biochemical cascades leading to apoptosis by paraquat may translate fruitful insights into potential neuroprotective therapies for PD. Therefore, the regulation of apoptosis with inhibitors targeted against these proximal signaling cascades can be further explored as it provides a new therapeutic approach for this debilitating disease.

## Figures and Tables

**Fig. (1):**
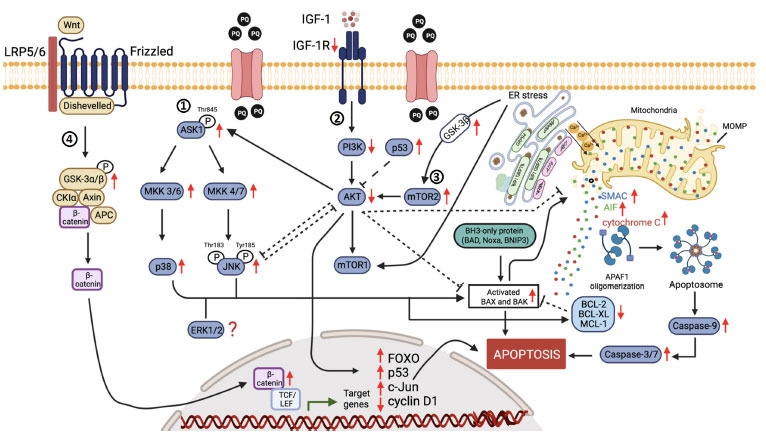
The upstream signal transduction pathways associated with paraquat-induced apoptosis. (**1**) MAPK, (**2**) PI3K/AKT, (**3**) mTOR, and (4) Wnt/b-catenin signaling pathways. **Abbreviations**: AIF, apoptosis-inducing factor; APAF1, apoptotic protease activating factor 1; APC, adenomatous polyposis coli; CKIα, casein kinase I alpha; ER, endoplasmic reticulum; ERK, extracellular signal-regulated kinase; FOXO, Forkhead box transcription factors of the class O; GSK-3, glycogen synthase kinase-3; IGF-1, insulin-like growth factor 1; IGF-1R, insulin-like growth factor 1 receptor; JNK, c-Jun N-terminal kinases; LRP5/6, low-density lipoprotein-related receptors 5 and 6; MAPK, mitogen-activated protein kinase; MCL-1, myeloid cell leukemia-1; MKK, mitogen-activated protein kinase kinase; MOMP, mitochondrial outer membrane permeabilization; mTOR, mammalian target of rapamycin; PI3K, phosphatidylinositol-4,5-bisphosphate 3-kinase; PQ, paraquat; SMAC, second mitochondria-derived activator of caspase; TCF/LEF, T-cell factor/lymphoid enhancer factor.

**Table 1:** Pharmacological effects of paraquat.

**References**	**Type of Exposure**	**Cell/Animal Model**	**Paraquat Doses**	**Route of ** **Administration**	**Treatment Duration**	**Effects**
Shukla *et al.*, 2014 [23]	*In vivo*	Male *Drosophila melanogaster* (*w^1118^*)	10 and 20 mM	Oral	12 and 24 h	↑ p-JNK, ↑ pFOXO/FOXO, ↑ cleaved caspase-3, ↑ TUNEL-positive cells
Wills *et al.*, 2012 [27]	*In vivo*	Male mice with a mixed C57BL/6 x 129S background (2-3 months)	10 mg/kg	Intraperitoneal	Twice a week for 6 weeks	↑ α-synuclein, ↑ p-tau, ↑ p-GSK-3β, ↑ mTOR, ↓ autophagic flux
Srivastav *et al.*, 2018 [41]	*In vivo*	Male *Drosophila melanogaster* (Oregon-R-P2)	20 mM	Oral	48 h	↑ p-JNK, ↑ cleaved caspase-3, ↑ Nrf2
Niso-Santano *et al.*, 2010 [51]	*In vitro*	Human neuroblastoma SH-SY5Y cells	25-750 μM	Not applicable	24 h	↓ ASK1, ↓ p-ASK1ser83, ↓ p-ASK1ser967, ↑ p-ASK1thr845, ↑ p-MKK3/6, ↑ p-MKK4/7, ↑ p-p38, ↑ p-JNK, ↑ p-c-Jun
Niso-Santano *et al.*, 2010 [53]	*In vitro*	Human neuroblastoma SH-SY5Y cells overexpressing wild-type ASK1	100 μM	Not applicable	24 h	↑ ER stress, ↑ autophagy
Ju *et al.*, 2019 [62]	*In vitro*	Human neuroblastoma SH-SY5Y cells	300 μM	Not applicable	24 h	↑ TUNEL-positive cells, ↑ p-p38, ↑ p-JNK, ↓ p-ERK, ↑ p-c-Jun, ↑ p-p53, ↑ BAD, ↑ BAX/BCL-2 ratio, ↑ cytochrome C, ↑ caspase-9, ↑ caspase-3, ↑ PARP, ↓ p-IGF1R, ↓ p-PI3K, ↓ p-AKT
Seo *et al.*, 2014 [70]	*In vitro*	Mouse NIH-3T3 embryonic fibroblasts	100-1000 μM	Not applicable	24 h	↑ p-Elk1, ↑ ERK1/2, ↑ cytochrome c, ↑ DNA fragmentation
Niso-Santano *et al.*, 2006 [71]	*In vitro*	Rat brain neuroblasts (E18 cells)	25 μM	Not applicable	24 h	↑ p-ERK1/2, ↑ p-JNK1/2, ↑ p-AKT
Yang *et al.*, 2018 [119]	*In vitro*	Human neuroblastoma SH-SY5Y cells	300 μM	Not applicable	24 h	↑ cleaved PARP, ↑cleaved caspase-3, ↑ β-catenin, ↑ p-GSK-3ɑ/β, ↓ cyclin D1, ↑ BAK, ↑ cytochrome C, ↑ DIABLO, ↑ HtrA2

**Abbreviations:** ASK1, apoptosis signal-regulating kinase 1; DIABLO, direct inhibitor of apoptosis-binding protein with low pI; ER, endoplasmic reticulum; ERK, extracellular signal-regulated kinase; FOXO, Forkhead box transcription factors of the class O; GSK-3, glycogen synthase kinase-3; HtrA2, high-temperature requirement-A serine peptidase-1; IGF-1R, insulin-like growth factor 1 receptor; JNK, c-Jun N-terminal kinases; MKK, mitogen-activated protein kinase kinase; mTOR, mammalian target of rapamycin; Nrf2, nuclear factor erythroid 2-related factor 2; PARP, poly (ADP-ribose) polymerase; PI3K, phosphatidylinositol-4,5-bisphosphate 3-kinase; TUNEL, terminal deoxynucleotidyl transferase dUTP nick end labelling.

## LIST OF ABBREVIATIONS

ASK1: Apoptosis Signal-regulating Kinase 1
DAT: Dopamine Transporter
ERK: Extracellular Signal-regulated Kinase
IGF-1: Insulin‐like Growth Factor 1
MOMP: Mitochondrial Outer Membrane Permeabilization
PD: Parkinson’s Disease
RNS: Reactive Nitrogen Species
ROS: Reactive Oxygen Species
RTK: Receptor Tyrosine Kinase
SAPK: Stress-activated Protein Kinase
UPR: Unfolded Protein Response
